# Estimating osteoporotic fracture risk following a wrist fracture: a tale of two systems

**DOI:** 10.1007/s11657-015-0218-3

**Published:** 2015-05-09

**Authors:** Karen Beattie, Jonathan Adachi, George Ioannidis, Alexandra Papaioannou, William D. Leslie, Ruby Grewal, Joy MacDermid, Anthony B. Hodsman

**Affiliations:** Department of Medicine, McMaster University, 501-25 Charlton Ave. East, Hamilton, ON L9N 1Y2 Canada; St. Peter’s Hospital, McMaster University, 88 Maplewood Ave, Hamilton, ON L8M 1W9 Canada; Departments of Medicine and Radiology, University of Manitoba, C5121-409 Tache Avenue, Winnipeg, MB R2H 2A6 Canada; Hand and Upper Limb Center, St. Joseph’s Health Care, Western University, 268 Grosvenor St., London, ON N6A 4L6 Canada; Osteoporosis and Metabolic bone Disease Program, Lawson Health Research Unit, St. Joseph’s Health Care, Western University, 268 Grosvenor St., London, ON N6A 4L6 Canada

**Keywords:** Osteoporosis, Distal radius fracture, Clinical practice guidelines, Fracture risk, FRAX

## Abstract

***Summary*:**

The WHO fracture risk assessment (FRAX) and Canadian Association of Radiologists and Osteoporosis Canada (CAROC) tools can both be used to determine an individual’s 10-year risk of osteoporotic fracture. However, these tools differ in their risk calculation. For participants <65 years with a wrist fracture, FRAX provides a lower fracture risk estimate than CAROC resulting in fewer decisions to initiate therapy.

**Purpose:**

The purpose of the current report is to compare fracture risk prediction rates using the CAROC and the FRAX® tools.

**Methods:**

Individuals ≥50 years with a distal radius fracture resulting from a fall from standing height or less were recruited from a single orthopedic clinic. Participants underwent a DXA scan of their lumbar spine and hip. Femoral neck (FN) bone mineral density (BMD) and fracture risk factors were used to determine each participant’s 10-year fracture risk using both fracture risk assessment tools. Participants were categorized as low (<10 %), moderate (10–20 %), or high (>20 %) risk. Stratified by age (<65 years, >65 years), the proportion of participants in each category was compared between the tools.

**Results:**

Analyses included 60 participants (mean age 65.7 ± 9.6 years). In those <65 years (*n* = 26), the proportion of individuals at low, moderate, and high risk differed between the FRAX and CAROC tools (*p* < 0.0001). FRAX categorized 69 % as low (CAROC 0 %) and 3 % as high (CAROC 12 %) risk. For individuals >65 years, almost all were at least at moderate risk (FRAX 79 %, CAROC 53 %), but fewer were at high risk using FRAX (18 vs. 47 %, *p* < 0.0003).

**Conclusion:**

For participants <65 years with a wrist fracture, FRAX provides a lower estimate of 10-year fracture risk than CAROC resulting in fewer decisions to initiate therapy. However, almost all participants >65 years were at moderate or high risk under both FRAX and CAROC and should at least be considered for pharmacotherapy.

## Introduction

Following publication of the 2010 clinical practice guidelines (CPG) for the diagnosis and management of osteoporosis in Canada [1], Osteoporosis Canada emphasized the need to evaluate future fracture risk in all older individuals who experience a major “fragility” fracture typically associated with osteoporosis (i.e., hip, clinical or morphometric vertebra, pelvis, and forearm or proximal humerus).

In Canada, two fracture risk prediction tools exist to determine an individual’s 10-year risk of osteoporotic fracture. The Canadian Association of Radiologists and Osteoporosis Canada (CAROC) tool stratifies women and men >50 years into three categories of osteoporotic fracture risk: low (<10 %), moderate (10–20 %), and high (>20 %) [2]. Baseline risk is assessed using age, sex, and femoral neck T-score. The presence of a prior fragility fracture after age 40 [3] or recent prolonged use of systemic glucocorticoids increases fracture risk into the next highest category independent of bone mineral density (BMD). The World Health Organization Fracture Risk Assessment (FRAX^©^) tool uses additional risk factors over and above those used in CAROC (e.g., parental history of hip fracture) [4]. Lumbar spine BMD is not considered in either tool.

Overall, observed fracture rates for Canadians are in close agreement to the rates predicted by both tools [5]. Osteoporosis Canada recommended the CAROC tool be adopted in preference to FRAX based on its relative simplicity and wider national availability. The CPG recommend that all high-risk individuals be offered pharmacotherapy, while those at moderate risk be regarded with some circumspection before initiating therapy and only considered if there are other risk factors and in accordance with patient preference. This is important in primary practice considering wrist fractures comprise 26–46 % of all observed skeletal fractures [6–11] and are the most common fragility fracture seen in fracture clinics in this age group.

Although a fragility fracture increases the risk for future fractures, there is evidence that wrist fractures are associated with significantly lower re-fracture rates in the following 5–10 years than is observed following other “osteoporotic” fractures (e.g., vertebra, hip, or proximal humerus), especially in younger individuals with non-osteoporotic BMD values [12]. Here, we compare the results of applying the CAROC and FRAX tools to 60 participants presenting to a single orthopedic clinic after a wrist fracture to assess differences in treatment directives resulting from their respective risk stratification.

## Methods

Individuals ≥50 years old presenting with a fragility fracture [13] of the distal forearm were recruited from the orthopedic clinic at St. Joseph’s Health Care, London, Ontario. Individuals previously diagnosed with osteoporosis by self-report or currently taking osteoporosis medication were excluded. Given that the study’s primary goal was to observe the response of family physicians to the patients’ fracture risk reported by CAROC or FRAX (prescribed treatment or not), participants who had not previously been diagnosed with osteoporosis or who had not been treated for osteoporosis were the target study population [14]. Subjects consented to enrolment in a clinical study designed to monitor treatment outcomes from their family physicians and were provided with BMD assessments. A trained research assistant collected baseline demographic information and information about fragility fractures after age 40 years. BMD testing was performed on a Lunar Prodigy Advance system (GE Healthcare, with a precision of ±0.01 g/cm^2^ at the femoral neck). Under current guidelines, only the femoral neck T-score was used for risk categorization. Osteophytic artifact due to degenerative disk disease, facet joint arthropathy, etc. formed part of the lumbar spine report structure if visually apparent on the densitometer images. All BMD reports were read by one trained clinician (ABH).

The purpose of the current report is to compare and contrast the risk categorization between the CAROC and FRAX tools when applied to all 60 participants. The Canadian reference population was used for the FRAX calculation. Each participant’s fracture risk was assessed using each tool. Incomplete information on parental history of hip fracture collected at study entry might have systematically underestimated future fracture risk calculated for FRAX. We address the question of whether the use of either tool in primary practice will lead to the same or discordant therapeutic decisions. This study was approved by the Institutional Review Board for Research involving Human Subjects at Western University and the Hamilton Integrated Research Ethics Board at McMaster University.

## Analyses

Baseline demographic data are reported as means (SD). Statistical differences between younger and older participants were defined by Student’s *t* test. Categorical differences in the proportion of participants assigned to the three risk categories between the CAROC and FRAX systems were evaluated by testing for marginal homogeneity using the Bhapkar test.

## Results

Baseline demographic data are shown for all participants stratified by age (Table 1). For all 60 participants, the mean age was 65.7 ± 9.6 years. Almost half (*n* = 26 [43 %]) were younger than 65 years, <20 % were men and 18 (30 %) recalled a parental history of either osteoporosis or fracture. Few were currently taking glucocorticoids or undergoing treatment for rheumatoid arthritis.

**Table 1:** Baseline characteristics for all participants and stratified by age

	All	<65 years	>65 years	*p* value^a^
Number of subjects	60	26	34	
Age, years	65.7 ± 9.6	56.7 ± 4.6	72.5 ± 6.0	<0.001
Female/male	49/11	21/5	28/6	
Fracture prior to current wrist fracture	2	2	0	
Family history of osteoporosis	18	9	9	
Current steroid therapy	2	0	2	
Rheumatoid arthritis	3	1	2	
FRAX patients				
Femoral neck BMD, g/cm^2^	0.85 ± 0.12	0.91 ± 0.16	0.81 ± 0.06	0.022
Femoral neck T-score	−1.2 ± 0.9	−0.8 ± 1.2	−1.5 ± 0.6	0.034
Lumbar spine BMD, L_2_–L_4_, g/cm^2^	1.15 ± 0.24	1.12 ± 0.24	1.17 ± 0.25	NS
Lumbar spine T-score	−0.5 ± 2.0	−0.7 ± 1.9	−0.3 ± 2.0	NS
CAROC patients				
Femoral neck BMD, g/cm^2^	0.87 ± 0.10	0.92 ± 0.11	0.82 ± 0.06	0.003
Femoral neck T-score	−1.0 ± 0.9	−0.5 ± 0.9	−1.4 ± 0.7	0.004
Lumbar spine BMD, L_2_–L_4_, g/cm^2^	1.13 ± 0.15	1.18 ± 0.18	1.08 ± 0.11	NS
Lumbar spine T-score	−0.6 ± 1.3	−0.2 ± 1.5	−0.98 ± 1.0	NS
FRAX, 10-year probability of future fragility fracture, %	12.5 ± 5.1	9.1 ± 4.0	15.2 ± 4.3	<0.001

*NS* not significant

^a^ *p* values represent comparisons between <65 years and >65 years

Femoral neck T-scores were not lower than expected for the age- and sex-matched reference population (Table 1). Femoral neck BMD was significantly (*p* < 0.001) higher in participants <65 years compared with those >65 years (T-score −1.1 ± 0.9 vs. −1.5 ± 0.6). Lumbar spine BMD was similar between groups, and T-scores were slightly higher than those at the femoral neck. This is likely explained by a high prevalence of measurement artifact due to osteophytic disease (nearly 80 % of cohort).

In applying the FRAX tool to all participants, the mean 10-year probability of future major osteoporotic fracture was 12.5 ± 5.1 %. Fracture probability was significantly lower in those <65 years than in those >65 years (9.1 ± 4.0 vs. 15.2 ± 4.3 %, *p* < 0.001) (Table 1).

Table 2 demonstrates the respective differences in risk category by the CAROC and FRAX systems based upon the proportion of participants at low, moderate, and high risk. Using FRAX, 19 (32 %) of all participants were at low risk while only 7 (12 %) were at high risk. Differences in the proportions of participants categorized according to their fracture risk were significant (*p* ≤ 0.001) whether analyzed for all ages combined, in younger or older participants.

**Table 2:** Fracture risk category for all participants and those younger and older than 65 years using FRAX and CAROC

	FRAX	CAROC
All subjects, *n* = 60^a^	N (%)	N (%)
Low	19 (32)	0
Moderate	34 (57)	41 (68)
High	7 (12)	19 (32)
Age <65 years, *n* = 26^a^		
Low	18 (69)	0
Moderate	7 (27)	23 (88)
High	1 (4)	3 (12)
Age > 65 years, *n* = 34^a^		
Low	1 (3)	0
Moderate	27 (79)	18 (53)
High	6 (18)	16 (47)

The proportion of subjects assigned to each of the three risk categories is significantly different between the FRAX and CAROC tools, both for all subjects, for those ≤65 years and for those >65 years (*p* < 0.001)

^a^ *p* < 0.001 for difference in risk categorization

In the 26 participants <65 years, FRAX categorized 18 (69 %) as low risk and only 1 (3 %) as high risk. None of the participants <65 years was low risk by CAROC due to an automatic increase of one risk category based upon the presence of a prior fragility wrist fracture. CAROC categorized 23 (88 %) as moderate and 3 (12 %) as high risk.

For participants >65 years, almost all were at least at moderate risk (FRAX 79 %, CAROC 53 %), but FRAX categorized fewer participants as high risk (18 vs. 47 %).

## Discussion

It is clear that the two fracture risk assessment tools available to Canadian physicians provide significantly different risk estimates for adults presenting with a wrist fracture, particularly in those <65 years. In the current study, more than half of wrist fractures occurred in individuals <65 years of age in whom average femoral neck T-scores were considerably higher than −2.5 and typically average for age. For this important group of individuals, the operating characteristics of FRAX differ significantly from CAROC. The FRAX algorithm provides a continuous assessment of future fracture risk and includes prevalent fragility fractures within the statistical modeling. Using FRAX, participants with prior fractures may still be at low risk of future fractures. On the other hand, CAROC does not predict a low risk in anyone presenting with a fragility fracture. Even if the measured BMD indicates excellent bone mass, the baseline low risk will be increased to moderate risk by the prevalent fracture. Thus, FRAX categorized two thirds of those <65 years as low risk—a group for whom the 2010 CPG advise no consideration for pharmacological intervention. Only 1 patient was high risk and thus a candidate for initiating therapy. The picture is very different when CAROC is used. All participants <65 years would at least be considered for pharmacotherapy, as they were at moderate (88 %) or high (12 %) risk.

The situation is less disparate in older individuals. In those >65 years, two and a half times as many were categorized as high risk by the CAROC tool, compared with FRAX, and would therefore be advised to initiate pharmacotherapy. However, all participants otherwise characterized as moderate risk by CAROC would also be similarly characterized by FRAX; again, these individuals should at least be considered for pharmacotherapy, depending on other risk factors and patient preferences.

It is now generally accepted that any prevalent fragility fracture is a marker of increased fracture risk. This was first clearly demonstrated for clinical and/or morphometric vertebral fractures [15, 16], but has been confirmed for all fragility fractures in epidemiological cohort studies and in large randomized controlled trials of new osteoporosis therapies in which individuals specifically at risk for fragility fractures were studied [3]. The risk of subsequent fracture after wrist fracture may, in part, be age- and gender-dependent, as demonstrated by [17] et al., with women <70 years of age having been shown to have no increase in subsequent hip fracture risk.

Hodsman et al. evaluated the risk of refracture in women presenting with a primary wrist fracture. In this study, the observed 10-year refracture rate across all ages (≥50 years) was 14.2 % (95 % CI, 11.9–16.5), consistently in the moderate risk category. This rate was significantly lower for individuals <65 years (compared to those >65 years) and for individuals with T-scores >−2.5 [12]. Thus, some individuals are clearly at low risk of refracturing after a primary wrist fracture and would not be advised to initiate treatment under the current CPG. This is important considering wrist fractures comprise up to 50 % of the clinical fragility fracture burden within the population.

Many individuals view their wrist fracture as an unfortunate result of a traumatic fall and are reluctant to have their accident “medicalised” to the point of starting an osteoporotic treatment. The FRAX tool allows the practitioner a more flexible environment in which to arrive at an informed decision with the patient around the initiation of therapy. FRAX may be the preferred tool as it will be more conservative in selecting individuals for pharmacotherapy.

This argument may be especially relevant for individuals <65 years presenting with a wrist fracture. Provided the wrist fracture is the first “fragility” fracture in adult life, and after confirming there is no evidence for morphometric vertebral compression fractures, the physician may consider such individuals at low risk for refracture. Regardless, planned surveillance with interval BMD monitoring should be the standard of care for all individuals with a wrist fracture in whom a conservative approach is adopted.

## Limitations

Results from this small study clearly demonstrate that the FRAX and CAROC fracture risk prediction tools provide different estimates of the likelihood of future “osteoporotic” fractures following a wrist fracture, particularly in individuals <65 years. However, this requires validation by applying each tool prospectively to BMD measurements obtained from population-based studies in which future fractures are documented in those presenting with primary wrist fractures.

## Conclusions

For individuals <65 years who have sustained a wrist fracture, the FRAX tool provides a lower 10-year fracture risk estimate than CAROC and appropriately results in fewer decisions to initiate therapy. However, almost all individuals >65 years are at moderate or high risk under both FRAX and CAROC and should at least be considered for pharmacotherapy.
